# Thrombus in Transit Through Patent Foramen Ovale Causing Splenic Infarction

**DOI:** 10.1016/j.jaccas.2025.103924

**Published:** 2025-07-09

**Authors:** Rana Rashwan, Judy Rizk, Ingy Etman, Anhar Zokailah, Zeyad Kholeif

**Affiliations:** a Cardiovascular Department, Mayo Clinic, Rochester, Minnesota, USA; b Alexandria Main University Hospital, Alexandria, Egypt

**Keywords:** case report, paradoxical embolization, patent foramen ovale, pulmonary embolism, splenic infarction, systemic embolization, thrombus in transit

## Abstract

**Background:**

Patent foramen ovale (PFO) is often asymptomatic but can cause paradoxical embolism. Although cryptogenic stroke is common, this case is unique due to splenic infarction and a visible thrombus in transit.

**Case Summary:**

A 30-year-old woman presented with acute dyspnea, hemoptysis, and left upper quadrant abdominal pain. Investigations revealed deep vein thrombosis complicated by pulmonary embolism and paradoxical embolization through PFO, leading to splenic infarction—a rare sequence of events.

**Conclusions:**

Screening for PFO should be considered in patients with venous thrombosis and cryptogenic embolism. Thrombus in transit through PFO presents a challenge due to the lack of consensus on management.

## Graphical Abstract

**Figure:**
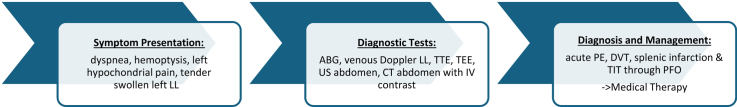

## Case Summary

**Figure 1:**
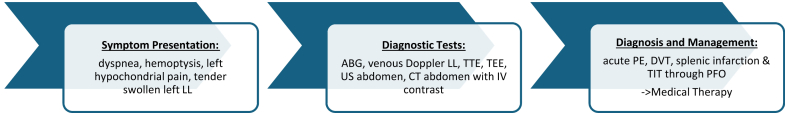
Clinical Snapshot: PFO and Paradoxical Embolism A patient presenting with dyspnea, hemoptysis, and left upper quadrant pain diagnosed as PE, DVT, and splenic infarction due to paradoxical embolism through a PFO.^1^ The figure outlines key symptoms, diagnostic evaluation, and management approach. ABG = arterial blood gas; CT = computed tomography; DVT = deep vein thrombosis; IV = intravenous; LL = lower limb; PE = pulmonary embolism; PFO = patent foramen ovale; TIT = thrombus in transit; TTE = transthoracic echocardiography; US = ultrasound.

A 30-year-old woman with a body mass index of 22 kg/m^2^ presented to the emergency department with acute worsening dyspnea and hemoptysis that had begun 2 days before (Figure 1). She also reported dull left upper abdominal pain starting 12 hours before presentation. The examination revealed tachypnea (respiratory rate of 28 breaths/min) and blood pressure of 100/60 mm Hg. Notably, her left lower limb was swollen and tender, and there was left hypochondrial abdominal tenderness.

**Take-Home Messages**

• Thrombus in transit through a PFO leading to paradoxical embolization is rare.
• Screening for PFO should be part of the evaluation for patients with venous thrombosis and unexplained arterial embolization.

The patient had a history of deep vein thrombosis (DVT) and pulmonary embolism (PE) twice, 5 and 3 years ago, respectively. She was taking a daily dose of 5 mg of warfarin but had neglected regular INR monitoring. Additionally, she had been using combined oral contraceptive pills for 3 years. There was no history of immobility, tobacco, or recreational drug use.

The blood cultures taken to rule out infective endocarditis returned negative. The work-up for hypercoagulable states, including factor V Leiden mutation and antiphospholipid syndrome, was also negative. Tests for disseminated intravascular coagulation showed no abnormalities.

Arterial blood gas analysis indicated hypoxia and respiratory alkalosis (O_2_: SaO_2_ 90% and CO_2_: 28 mm Hg). An electrocardiogram showed sinus tachycardia, and the international normalized ratio (INR) was 1.8, below the therapeutic range for DVT/PE treatment. A venous Doppler scan confirmed DVT in the left lower limb. Given the high suspicion of PE, a transthoracic echocardiography was performed,^2^ revealing right-sided dilation and other findings indicative of PE (Video 1). A mobile mass was identified in the right atrium, leading to a transesophageal echocardiography (TEE) that confirmed a patent foramen ovale (PFO) with a “thrombus in transit” (Video 2). An abdominal ultrasound indicated splenic infarction, confirmed by computed tomography.

The treatment options included urgent surgical embolectomy and PFO closure,^3^ but the patient opted for nonsurgical management. She was admitted to the cardiac care unit for close monitoring, parenteral anticoagulation with unfractionated heparin, supplemental oxygen, and intravenous analgesia. After 48 hours of stability, she transitioned to rivaroxaban (15 mg twice a day for 21 days then 20 mg daily) and received an inferior vena cava filter to prevent further thromboembolism.

## Follow-up

A follow-up transthoracic echocardiography after 5 days showed a reduction in the thrombus size. The patient was advised to discontinue oral contraceptive pills to mitigate her DVT risk. She was discharged after 10 days with a scheduled follow-up TEE in 1 month to evaluate the thrombus resolution and her candidacy for percutaneous PFO closure. The follow-up TEE indicated that the thrombus had been resolved. She remains on rivaroxaban while awaiting the PFO closure procedure.

## Funding Support and Author Disclosures

The authors have reported that they have no relationships relevant to the contents of this paper to disclose.

## Appendix

Video 1 Trans thoracic echocardiography 4-chamber apical view showing the mobile mass in the right atrium related to the interatrial septum.

Video 2 Transesophageal echocardiography showing a tunnel-like patent foramen ovale harboring a mobile mass “thrombus in transit” straddling the patent foramen ovale with mobile parts in the left and right atria.
